# Mast Cells Are Activated by *Streptococcus pneumoniae In Vitro* but Dispensable for the Host Defense Against Pneumococcal Central Nervous System Infection *In Vivo*

**DOI:** 10.3389/fimmu.2018.00550

**Published:** 2018-03-19

**Authors:** Johanna Fritscher, Daniel Amberger, Susanne Dyckhoff, Jan Philipp Bewersdorf, Ilias Masouris, Stefanie Voelk, Sven Hammerschmidt, Helga Maria Schmetzer, Matthias Klein, Hans-Walter Pfister, Uwe Koedel

**Affiliations:** ^1^Department of Neurology, University Hospital, Ludwig-Maximilians-University, Munich, Germany; ^2^Department of Internal Medicine III (Oncology), University Hospital, Ludwig-Maximilians-University, Munich, Germany; ^3^Department of Molecular Genetics and Infection Biology, Interfaculty Institute for Genetics and Functional Genomics, Center for Functional Genomics of Microbes, Ernst Moritz Arndt University Greifswald, Greifswald, Germany

**Keywords:** mast cell, bacterial meningitis, innate immunity, *Streptococcus pneumoniae*, mouse model

## Abstract

Mast cells reside on and near the cerebral vasculature, the predominant site of pneumococcal entry into the central nervous system (CNS). Although mast cells have been reported to be crucial in protecting from systemic bacterial infections, their role in bacterial infections of the CNS remained elusive. Here, we assessed the role of mast cells in pneumococcal infection *in vitro* and *in vivo*. In introductory experiments using mouse bone marrow-derived mast cells (BMMC), we found that (i) BMMC degranulate and release selected cytokines upon exposure to *Streptococcus pneumoniae*, (ii) the response of BMMC varies between different pneumococcal serotypes and (iii) is dependent on pneumolysin. Intriguingly though, apart from a slight enhancement of cerebrospinal fluid (CSF) pleocytosis, neither two different mast cell-deficient *Kit* mutant mouse strains (WBB6F1-*Kit*^W/Wv^ and C57BL/6 *Kit*^W-sh/W-sh^ mice) nor pharmacologic mast cell stabilization with cromoglycate had any significant impact on the disease phenotype of experimental pneumococcal meningitis. The incomplete reversal of the enhanced CSF pleocytosis by local mast cell engraftment suggests that this phenomenon is caused by other c-Kit mutation-related mechanisms than mast cell deficiency. In conclusion, our study suggests that mast cells can be activated by *S. pneumoniae in vitro*. However, mast cells do not play a significant role as sentinels of pneumococcal CSF invasion and initiators of innate immunity *in vivo*.

## Introduction

Bacterial meningitis is among the top 10 causes of infection-related deaths worldwide, and many survivors suffer from permanent neurologic and otologic sequelae (1, 2). The most frequent causative agent in the Western world is *Streptococcus pneumoniae* (the pneumococcus), being responsible for more than half of all cases (1, 2). Pneumococcal infection of the meninges generates a strong inflammatory reaction, which contributes substantially to brain damage in this disease (2). The immune response is initiated by the recognition of pathogen-associated molecular patterns on invading pathogens by host pattern recognition receptors (PRRs) like toll-like receptors (TLR). TLR engagement induces myeloid differentiation primary response gene (MyD) 88-dependent production of a vast array of pro-inflammatory host factors including cytokines, chemokines, and complement factors (2). As a consequence, large numbers of neutrophils are recruited into the cerebrospinal fluid (CSF)-filled leptomeningeal space. Rapid recruitment of neutrophils to sites of infection is required for an effective host defense against invading pathogens (3). However, their many defense mechanisms that destroy or digest pathogens can also be deleterious to host tissue. Over the past decades, evidence has accumulated that neutrophils are indeed major effectors of tissue damage in pneumococcal meningitis (4–6). Since they are virtually absent in normal CSF, other immunocompetent cells might function as sentinels of bacterial CSF invasion and initiators of the host immune response.

Among the potential candidates to act as sentinels are mast cells. They are typically found not only in the meninges and choroid plexus but also within the brain parenchyma, particularly in the thalamic–hypothalamic region (7, 8). Mast cells generally reside on and near the vasculature, the predominant site of pneumococcal entry into the CSF (9). *In vitro*, mast cells, namely, the rat RBL-2H3 cell line, were found to respond by degranulation upon exposure to *S. pneumoniae* (10). Moreover, human lung mast cells and the human mast cell lines HMC-1 and LAD exhibited direct antimicrobial activity against *S. pneumoniae*, presumably through a pneumolysin (PLY)-induced release of the antimicrobial peptide LL-37 (11). Surprisingly, *in vivo*, in an experimental pneumococcal pneumonia model, mast cell-deficient *Kit*^W-sh/W-sh^ mice showed reduced bacterial outgrowth, less inflammation, and prolonged survival as compared with wild-type (WT) mice (12). This finding contrasts with the until-recently widely held concept that mast cells are crucial in protecting the host from bacterial infections (13, 14). This perspective goes back to two seminal reports that demonstrated, by using mast cell-deficient *Kit*^W/Wv^ mice, that mast cells are crucial for the survival of mice subjected to cecal ligation and puncture (CLP)-induced sepsis or *Klebsiella pneumoniae* peritonitis (15, 16). The protective effect was linked to mast cell-mediated promotion of neutrophil recruitment to sites of infection through their release of pro-inflammatory mediators (17, 18). Subsequently, numerous reports were published corroborating this initial observation in various experimental infectious disease models including, for instance, *Citrobacter rodentium*-induced colitis (19), experimental cystitis due to *Escherichia coli* infection (20), *Staphylococcus aureus* peptidoglycan-triggered diarrhea (21), *Salmonella minnesota* lipopolysaccharide-induced peritonitis (22), and *Pseudomonas aeruginosa* pneumonia (23), or encephalomyocarditis viral myocarditis (24). However, recent studies using two or more mast cell-deficient *Kit* mutant mouse strains and/or mutant mouse strains with unperturbed c-Kit function revealed a more complicated picture: depending on the nature of the mutation resulting in a mast cell deficiency as well as the type and severity of infection, mast cells can have no effect, aggravate, or attenuate inflammation and infectious disease severity (25–28). For example, mast cell engraftment enhances survival after moderately severe CLP in both WBB6F1-*Kit*^W/Wv^ mice and C57BL/6 *Kit*^W-sh/W-sh^ mice, but it either has no statistically significant effect on survival in WBB6F1-*Kit*^W/Wv^ mice or even impairs survival in C57BL/6-*Kit*^W-sh/W-sh^ mice (25). Thus, a conclusion for an absolute mast cell role in (infectious) diseases requires evidence from more than one mutant mouse strain (26, 29). Also of note, mast cell phenotype and function are direct consequences of the local microenvironment and have a marked influence on their ability to specifically recognize and respond to pathogens (30). Except for one study, which described a normal viral clearance, but less vigorous brain inflammation in mast cell-deficient mice as compared with WT mice when subjected to intracerebral Sindbis virus infection (31), investigations on the functional significance of mast cells in central nervous system (CNS) (especially bacterial) infections were lacking.

To provide some insight into the role of mast cells in bacterial CNS infections, here we assessed the phenotype of two different mast cell-deficient *Kit* mutant mouse strains and also the treatment effect of the so-called “mast cell stabilizer” cromoglycate in a well-established mouse model of pneumococcal meningitis (which represents a common and serious form of bacterial CNS infection).

## Materials and Methods

### Animal Experimentation

All procedures were approved by the Committee on the Ethics of Animal Experiments of the Government of Upper Bavaria (Permit numbers 55.2-1-54-2531-67-99, -125-13) and carried out in accordance with the Principles of Laboratory Animal Care (European Commission Directive 2010/63/EU), the German Animal Welfare Act, and the ARRIVE guidelines (32). All experiments were conducted on age-matched male, 10- to 16-week-old mice. All efforts were made to minimize animal suffering and the number of animals used (8–12 mice per group, based on power calculations at 80% power and significance level of 5%). C57BL/6 mice (*n* = 30 in total), *Kit* mutant WBB6F1-*Kit*^W/Wv^ mice (*n* = 16, one mouse had to be euthanized due to neurologic abnormalities related to the meningitis induction procedure) and their congenic littermates WBB6F1-*Kit*^+/+^ (*n* = 12) and *Kit* mutant C57BL/6 *Kit*^W-sh/W-sh^ mice (*n* = 25), and their WT controls C57BL/6 *Kit*^+/+^ (*n* = 11; one mouse developed clinical signs requiring euthanasia) were purchased from Charles River Germany (Sulzfeld, Germany; the exclusive distributor of imported JAX^®^ mice strains in Germany). *Kit*^W/Wv^ mice carry one allele (W) with a point mutation that produces a non-functional truncated receptor and another allele (W-v) that encodes a mutation in the kinase domain. They are profoundly mast cell deficient (33). *Kit*^W-sh/W-sh^ mice, in turn, have an inversion upstream of the *Kit* gene that leads to a selective reduction of Kit expression and hence severe mast cell deficiency (34, 35). Both mouse strains have white spotted or all-white coats while their mast cell-sufficient congenic littermates have dark coat, preventing allocation concealment and blinding during assessment of clinical outcome. Before and after meningitis induction, mice were housed in their home cages in a temperature-controlled environment, with a 12-h light dark cycle and were given access to food and water *ad libitum*.

### Induction of Pneumococcal Meningitis and Clinical Scoring

A well-characterized mouse model was used in this study (6, 36). Briefly, adult mice were weighed, and their body temperature was taken. Then, they were clinically examined and scored. Clinical scoring compromised (i) a beam balancing test, (ii) a postural reflex test, and (iii) monitoring of the presence of piloerection, seizures or reduced vigilance. The healthy mouse score was set to 0 points, while 13 points were attributed to terminally ill mice which were euthanized according to ethics guidelines. After clinical examination, bacterial meningitis was induced by intracisternal (i.c.) injection of 1 × 10^5^ colony-forming units (cfu) *S. pneumoniae* serotype 2 (D39 strain) under short-term anesthesia with isoflurane. Controls were i.c. injected with phosphate-buffered saline (PBS). Eighteen hours later, mice were weighed, scored clinically, and temperature was measured again. After anesthesia with ketamine/xylazine, a catheter was placed into the cisterna magna. Through it, CSF was sampled for measurement of CSF interleukin (IL)-1β concentrations and white blood cell counts. Subsequently, blood samples were drawn by transcardial puncture. Deeply anesthetized mice were perfused with ice-cold heparin-containing PBS, and thereafter the brains (including cerebella) were removed and further processed for microbiological and histological analyses.

### Determination of Bacterial Titers in Blood and Brain

Cerebella were dissected and homogenized in sterile saline. Blood samples and cerebellar homogenates were diluted serially in sterile saline, plated on blood agar plates, and cultured for 24 h at 37°C with 5% CO_2_.

### Brain Cytokine Expression Pattern

Mice brains were screened for 32 cytokines using a commercially available cytokine antibody array (Mouse Cytokine Array C2 from RayBiotech Inc., USA). Detailed information about this array including antibody list, sensitivity data, and experimental protocol can be obtained at the supplier’s website.^1^ Briefly, 30 µm thick brain sections were homogenized in lysis buffer (10 mM HEPES at pH 7.9, 10 mM KCl, 1.5 mM MgCl_2_, and a mixture of protease inhibitors, including phenyl-methylsulfonyl fluoride, aprotinin, leupeptin, and pepstatin A), then centrifuged at 12,000 rpm for 15 min at 4°C. Protein concentrations were determined in supernatants using Nanoquant assay (Carl Roth GmbH, Karlsruhe, Germany), and equal protein amounts of 8 mice brains per group were pooled. A total of 1,500 µg pooled protein was applied per array membrane and handled according to the manufacturer’s instructions. For evaluation, membranes were digitized and analyzed as described previously (37).

### Measurement of Brain IL-1β

Mouse brain IL-1β concentrations were determined by ELISA according to the manufacturer’s instructions (R&D Systems, Wiesbaden-Nordenstadt, Germany).

### Gene Array Analysis

The relative mRNA expression of cytokines, chemokines, and some related inflammatory genes was analyzed with a pathway-specific oligonucleotide microarray. The mouse inflammatory cytokines and receptor Oligo GEArray^®^ (OMM-011; Superarray Inc., Bethesda, MD, USA) contain 112 inflammatory cytokines and receptors genes, and different housekeeping genes. Detailed information about this oligonucleotide array including description of gene probes, experimental protocol, and data analysis method can be obtained at the supplier’s website.^2^ In brief, 3 µg of total RNA that was extracted from frozen brain sections with TRIzol^®^ reagent (Invitrogen) and pooled from brain extracts of eight mice per group was reverse transcribed. Then, biotin-labeled cRNA was synthesized from cDNA with the use of the TrueLabeling-AMP™ Linear RNA Amplification Kit (Superarray). cRNA was purified using the ArrayGrade cRNA Cleanup Kit (Superarray), quantified, and hybridized overnight to inflammatory cytokine and receptor gene-specific probes that were spotted on the GEArray membranes. After incubation with streptavidin–AP conjugate, the array image was developed with CPD-Star chemiluminescent substrate (Superarray), recorded with X-ray film, scanned into raw data and analyzed by TINA 2.08e software (Raytest) (37). To test the validity of the method, the brain mRNA expression of cytokines, namely, Il-1β, IL-33, and IL-6, were also examined by RT-PCR according to the methods described previously (38).

### Experimental Groups

To characterize the role of mast cells in the pathobiology of pneumococcal meningitis, we compared the disease phenotypes of two different mast cell-deficient c-*Kit* mutant mouse strains, namely, WBB6F1-*Kit*^W/Wv^ mice (*n* = 12) and C57BL/6 *Kit*^W-sh/W-sh^ mice (*n* = 8) with that of their respective control strains (*n* = 12 and *n* = 10, respectively). For mast cell engraftment studies, 4-week-old C57BL/6 *Kit*^W-sh/W-sh^ mice (*n* = 6) were reconstituted i.c. with 10^6^ bone marrow-derived mast cells (BMMC; for preparation, see below) in 50 µl PBS through transcutaneous injection under short-term isoflurane anesthesia. Reconstitution was confirmed by toluidine blue staining of brain sections. In additional experiments, we evaluated the effect of the pharmacologic mast cell stabilizer cromoglycate (*n* = 10) in C57BL/6 mice with pneumococcal meningitis. Controls (10 C57BL/6 mice, 3 WBB6F1-*Kit*^W/Wv^ mice, and C57BL/6 *Kit*^W-sh/W-sh^ mice each) received the vehicle (PBS) alone. Cromoglycate was given i.c. (at a dosage of 75 µg/mouse) because it only minimally crosses the BBB (39, 40).

### Toluidine Blue Staining of Brain Section

For determination of mast cell numbers in control and infected brains, brain hemispheres were fixed in 10% formalin and embedded in paraffin. Brains were cut coronally into 5 µm thick sections using a microtome and stained with acidic toluidine blue, according to standard protocols (41). The numbers of brain mast cells were determined microscopically at 200× magnification in 10 systematically selected brain sections containing lateral ventricles and the dentate gyrus and given as *n* per square meter. All counts were carried out on coded slides by a single observer.

### Cell Culture Experiments

Mouse mast cells (BMMC) were prepared from bone marrow cells isolated from femora, as described by Jensen et al. (42) with slight modifications. Briefly, femora were flushed with Hanks’ balanced salt solution, and the cell suspension was forced through a 70-µm mesh. Collected cells were resuspended in complete mast cell medium containing RPMI1640, 10% heat-inactivated fetal bovine serum (FBS), 2 mM glutamine, 1 mM sodium pyruvate, 50 µM 2-mercaptoethanol, 100 U/ml penicillin, 100 µg/ml streptomycin, 10 ng/ml recombinant murine IL-3, and 10 ng/ml recombinant murine stem cell factor (SCF; both from Peprotech, Hamburg, Germany), and cultured at 37°C in 5% CO_2_. Cells were transferred periodically to fresh flasks to remove adherent cells, and maintained for 4–6 weeks to allow mast cell differentiation. At this time, more than 96% of cells expressed the mast cell markers c-Kit (CD117) and Fc-epsilon receptor I, as determined by flow cytometry analysis.

### Mast Cell Degranulation Assay

Bone marrow-derived mast cells were washed thrice with PBS, re-suspended in HEPES buffer (pH 7.4, 10 mM HEPES, 137 mM NaCl, 2.7 mM KCl, 0.4 mM Na_2_HPO_4_, 5.6 mM glucose, 1.8 mM CaCl_2_, 1.3 mM MgSO_4_, and 0.04% bovine serum albumin) and seeded in a 96-well microtiter plate at a density of 10^5^ cells/100 μl, followed by stimulation with (penicillin–streptomycin-lysed) *S. pneumoniae* or its vehicle solution (Todd’s Hewitt broth plus 0.5% yeast extract, THY) for 30 min at 37°C. The stimulation protocols included (i) different concentrations of *S. pneumoniae* serotype 2, (ii) additional *S. pneumoniae* serotypes, namely, a PLY-deficient isogenic D39 mutant (D39Δ*ply*) as well as a serotype 3, 7F, and 19A, (iii) different concentrations of recombinant PLY [expressed and purified as described previously (43)], (iv) selected sets of pharmacologic inhibitors, including anti-TLR2- and anti-TLR4-neutralizing antibodies [clones T2.5 and 1A6, from Hycult Biotech, Germany, and NovImmun SA, Switzerland, respectively, each at a dosage of 25 µg/ml (44)], the endosomal TLR antagonist chloroquine (20 µg/ml, Sigma-Aldrich GmbH, Germany) (45), and cromoglycate (0.1, 1, and 10 mM, Sigma-Aldrich GmbH), and (v) BMMC derived from mouse strains with genetic deficiencies in the TLR signaling pathway, namely, *MyD88/TRIF^−/−^* mice (resulting in a complete loss of TLR signaling), *3d/Tlr2/4^−/−^* (lacking TLR2 and TLR4 expression besides carrying a neutralizing missense mutation in UNC93b1 named 3d, leading to abrogation of TLR3, TLR7, TLR9, TLR11, TLR12, and TLR13 function), and TLR2/3/4/7/9^−/−^ mice, as well as BMMC derived from mice lacking apoptosis-associated speck-like protein (ASC^−/−^) and thus *S. pneumoniae*-induced caspase-1 activation and IL-1β/IL-18 secretion (46, 47) (femora from all these mouse strains were kindly provided by Prof. Carsten Kirschning, University of Duisburg-Essen, Germany). The calcium ionophore A23187 (1 µM; Sigma-Aldrich) was used as a positive control (PC). At the end of the stimulation period, microtiter plates were centrifuged, supernatants were collected, and cell pellets were solubilized in HEPES buffer supplemented with 0.1% Triton-X. Then, the β-hexosaminidase activity in cell lysates and supernatants were determined as previously described by Kuehn et al. (48). The percentage of β-hexosaminidase release, indicating the extent of degranulation, was calculated as the ratio of absorbance of the supernatant to the sum of absorbance in the supernatant and cell lysate, multiplied with 100.

### Mast Cell Stimulation and Measurement of Cytokine Release

Bone marrow-derived mast cells were washed thrice with PBS, resuspended in serum-, IL-3- and SCF-free mast cell medium (containing nutridoma-SP instead of FBS), and seeded in a 96-well microtiter plate at a density of 2 × 10^5^ cells/200 μl. After 1 h of rest, BMMC were stimulated with *S. pneumoniae* according to the protocol of the degranulation experiments. Six hours post pneumococcal challenge supernatants were harvested and assayed for cytokines. Controls received THY instead of *S. pneumoniae*. For screening purposes, supernatants from control and *S. pneumoniae*-challenged cells were subjected to a mouse Multi-Analyte ELISA Array Kit (M-005a; Qiagen, Hilden, Germany). In all other experiments, supernatants were assessed for the presence of mouse IL-6 and CCL2 using mouse DuoSet ELISA Kits (R&D Systems, Bio-Techne, Wiesbaden, Germany). Moreover, the LDH activity, a marker of cell damage, was determined in supernatants (S), centrifuged supernatants of control cells after lysis with Triton X-100 (PC), and in control medium [negative control (NC)] using a colorimetric assay kit (BioVision, Biocat GmbH, Heidelberg, Germany). Cytotoxicity was calculated as percentage LDH release by the ratio of (S − NC/PC − NC) − 100.

### Statistical Analysis

The principal statistical test was one-way analysis of variance and subsequent Bonferroni *post hoc* tests. Differences were considered significant at *P* < 0.05. Data are displayed as means ± SD.

## Results

### *S. pneumoniae* Induces Degranulation and Selected Cytokine Production of Murine BMMC *In Vitro*

**Figure 1:**
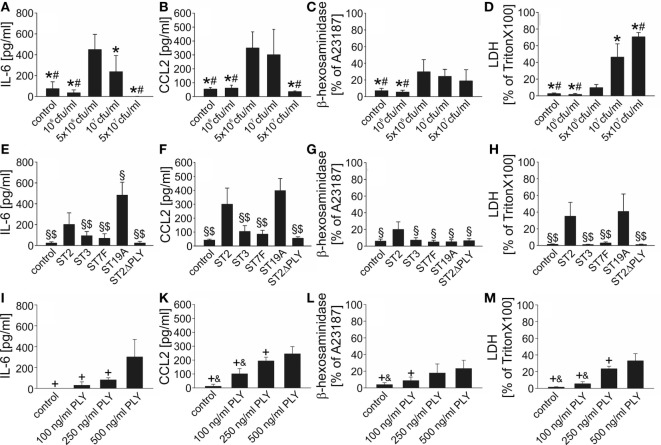
Mouse bone marrow-derived mast cells (BMMC) are activated upon exposure to *Streptococcus pneumoniae in vitro*. To get first insight into the role of mast cells in *S. pneumoniae* infection, we analyzed responsiveness of mouse BMMC toward pneumococcal challenge using different *S. pneumoniae* concentrations **(A–D)**, serotypes **(E–H)**, and different recombinant pneumolysin (PLY) concentrations **(I–M)**. Supernatants were collected 6 h after challenge, and interleukin (IL)-6 **(A,E)**, CCL2 **(B,F)**, β-hexosaminadase **(C,G)**, and LDH **(D,H)** contents were analyzed by standard assays. For β-hexosaminadase release measurements, the calcium ionophore A23187 served as positive control (PC). For LDH determinations, BMMC lysed by Triton X-100 were used as PCs. Controls received THY instead of *S. pneumoniae*. Abbreviations: ST, serotype; cfu, colony forming units. All experiments were—at least—carried out thrice in duplicates. Data are given as means ± SD. *P < 0.05, compared with 5 × 10^6^ cfu/ml *S. pneumoniae* ST2; ^#^*P* < 0.05, compared with 10^7^ cfu/ml *S. pneumoniae* ST2; ^§^*P* < 0.05, compared with 10^7^ cfu/ml *S. pneumoniae* ST2; ^$^*P* < 0.05, compared with 10^7^ cfu/ml *S. pneumoniae* ST19A; ^+^*P* < 0.05, compared with 500 ng/ml PLY, and ^&^*P* < 0.05, compared with 250 ng/ml PLY, using ANOVA and Student–Newman–Keuls test for *post hoc* analysis.

Data on the impact of *S. pneumoniae* on cultured mast cells are scarce and inconsistent: live (but not heat-killed) *S. pneumoniae* (serotype 2; D39 strain) triggered mast cell degranulation without concomitant cytokine (TNF-α and of IL-6) release in a rat mast cell line (10), whereas exposure of human lung mast cells and mast cell lines to the identical pneumococcal strain led to leukotriene C4 and antimicrobial peptide LL-37 release without histamine [a degranulation marker (49)] and cytokine liberation. To get a first hint into the responsiveness of murine BMMC to pneumococcal challenge, we stimulated 4- and 6-week-old mast cells with antibiotic-lysed *S. pneumoniae* (serotype 2; D39) and analyzed supernatants for the presence of 12 different cytokines using a Multi-Analyte ELISA Array Kit. Six hours post challenge, substantially elevated levels of IL-6, CCL2, CCL3, and CCL4 (but not IL-1β, TNF-α, IFN-γ, IL-12. IL-17A, IL-4, IL-10, and TGF-β_1_) were detectable in supernatants from stimulated BMMC as compared with control cells (irrespective of the mast cell culture duration; data not shown). Next, we investigated the concentration and serotype dependence of mast cell activation upon *S. pneumoniae* stimulation. The production of cytokines (we selected two out of the four alternatives presented by the Multi-Analyte ELISA investigations, namely, IL-6 and CCL2) was significantly increased at D39 infectious doses of 5 × 10^6^ and 1 × 10^7^ cfu/ml, but neither at lower nor at higher bacterial concentrations (Figures 1A,B). Quite similar results were obtained with the β-hexosaminidase degranulation assay (Figure 1C). The decline in cytokine and β-hexosaminidase release observed at higher bacterial concentrations is probably due to a substantial cytotoxicity of *S. pneumoniae* (serotype 2) on BMMC, as indicated by the detection of high LDH in the supernatant (Figure 1D). Furthermore, cytokine production and degranulation varied substantially between mast cells stimulated with different pneumococcal serotypes (Figures 1E–H): IL-6 and CCL2 production was observed only after exposure of mast cells to serotype 2 and 19A, but not to serotype 3 and 7F pneumococci. Mast cell degranulation occurred solely after challenge with the serotype 2 strain. Of note, genetic depletion of PLY resulted in a complete loss of the mast cell activating potency of the serotype 2 strain D39Δ*ply* (Figures 1E–H) and recombinant PLY on its own was able to induce cytokine production and degranulation of BMMC (Figures 1I–M). All in all, our data propose that mouse BMMC respond to *S. pneumoniae* infection with the release of selected host factors, and the BMMC response varies between serotypes and is largely dependent on the presence of PLY.

### Mast Cell Activation by *S. pneumoniae* Does Not Involve TLR Signaling

**Figure 2:**
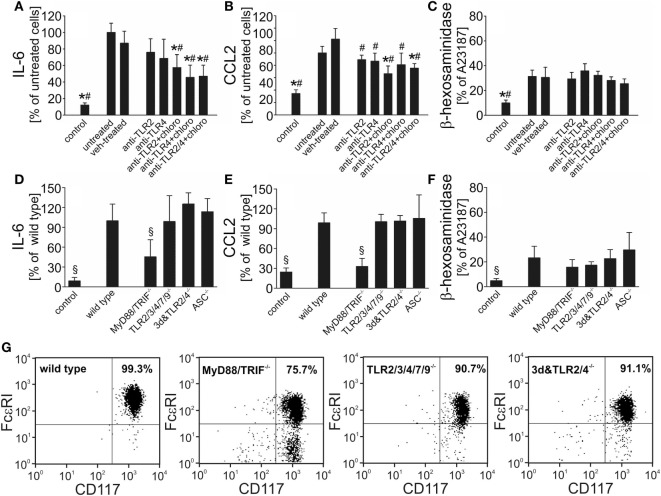
Bone marrow-derived mast cells (BMMC) activation in response to *Streptococcus pneumoniae* challenge occurs independently of toll-like receptors (TLR) signaling. To evaluate the role of TLR in *S. pneumoniae*–BMMC interaction, we assessed the responsiveness of BMMC to pneumococcal challenge (10^7^ cfu/ml) in the absence or presence of different TLR antagonists (including blocking antibodies directed against TLR2 or TLR4 and the endosomal TLR antagonist chloroquine). BMMC from mice with deficiencies in TLR signaling (including MyD88/TRIF-, TLR2/3/4/5/7/9-, and 3d&TLR2/4-deficent mice) or in the inflammasome component ASC were used in additional experiments. Supernatants were collected 6 h after challenge, and interleukin (IL)-6 **(A,D)**, CCL2 **(B,E)**, and β-hexosaminadase **(C,F)** levels were analyzed by standard assays. To determine the differentiation state of BMMC, cells sampled 6 weeks after the start of culture were subjected to immunophenotyping by flow cytometry using FcεRI and CD117 antibodies **(G)**. All experiments were—at least—carried out twice in triplicates. Data are given as means ± SD. *P < 0.05, compared with untreated, stimulated BMMC; ^#^*P* < 0.05, compared with vehicle (combination of isotype antibodies plus sterile deionized water, the vehicle of chloroquine)-treated, stimulated BMMC; and ^§^*P* < 0.05, compared with stimulated wild-type BMMC, using ANOVA and Student–Newman–Keuls test for *post hoc* analysis.

Recent investigations in cultured macrophages suggest involvement of TLR2, TLR4, and TLR9 in pneumococcal sensing *in vitro* (2). These TLRs have also been identified on and/or in murine BMMC (50). To assess their role in *S. pneumoniae*–BMMC interaction, we first stimulated BMMC with serotype 2 pneumococci in the absence or presence of different TLR antagonists. Supplementation of the cell culture medium with the endosomal TLR antagonist chloroquine, but not with neutralizing antibodies directed against TLR2 or TLR4, resulted in a significant inhibition of *S. pneumoniae*–induced IL-6 and CCL2 production, but had no effect on β-hexosaminidase release (Figures 2A–C). The combined addition of chloroquine and anti-TLR2/4 antibodies showed no further effect on *S. pneumoniae*-triggered cytokine release. Since chloroquine is known to exert other immunomodulatory effects than inhibition of endosomal TLR signaling (51, 52), we conducted additional experiments using BMMC from mice with profound deficiencies in TLR signaling. Upon exposure to *S. pneumoniae, MyD88/TRIF^−/−^* BMMC released significantly lower amounts of IL-6 and CCL2 (but not β-hexosaminidase) into the cell culture supernatant as compared with WT BMMC (Figures 2D–F). Surprisingly, BMMC obtained from *3d/Tlr2/4^−/−^* mice lacking TLR2 and TLR4 expression besides carrying a neutralizing missense mutation in UNC93b1 named 3d (leading to abrogation of TLR3, TLR7, TLR9, TLR11, TLR12, and TLR13 function), and from TLR2/3/4/7/9^−/−^ mice exhibited no difference to WT BMMC in respect to both, cytokine and β-hexosaminidase release, arguing against an involvement of TLR in *S. pneumoniae*–induced BMMC activation. Of note, flow cytometric immunophenotyping analysis revealed an impaired differentiation of *MyD88/TRIF^−/−^* bone marrow cells into mast cells, as compared with WT, but also *3d/Tlr2/4^−/−^* and TLR2/3/4/7/9^−/−^ cells: for instance, after 6 weeks of culture, only 75% of *MyD88/TRIF^−/−^* BMMC expressed the mast cell markers c-Kit and Fc-epsilon receptor I, whereas 99, 91, and 91% of WT, *3d/Tlr2/4^−/−^*, and TLR2/3/4/7/9^−/−^ cells were c-Kit—and Fc-epsilon receptor I—positive, respectively (Figure 2G). As a consequence, the number of BMMC was substantially lower in the *MyD88/TRIF^−/−^* group as compared with all other experimental groups, possibly explaining the reduction in IL-6 and CCL2 levels in the supernatants from *MyD88/TRIF^−/−^* BMMC cultures. Since MyD88 also acts as an adapter in the signaling cascade of IL-1 family cytokines, we additionally assessed the phenotype of *ASC^−/−^* BMMC following exposure to *S. pneumonia*e. ASC is a critical factor for inflammasome formation and subsequent caspase-1 activation and IL-1 family cytokine secretion (47, 53, 54). The differentiation behavior of *ASC^−/−^* BMMC was equal to that of WT cells: 97% of *ASC^−/−^* cells expressed the mast cell markers c-Kit and Fc-epsilon receptor I after 6 weeks of culture. Moreover, *ASC^−/−^* BMMC exhibited no difference to WT BMMC in respect to both, cytokine and β-hexosaminidase release. Thus, (autocrine) IL-1 signaling is unlikely to contribute to the phenotype of MyD88/TRIF^−/−^ BMMC (Figures 2D–F).

### The Number of Metachromatically Stained Mast Cells Is Reduced in the Brain During Pneumococcal Meningitis

**Figure 3:**
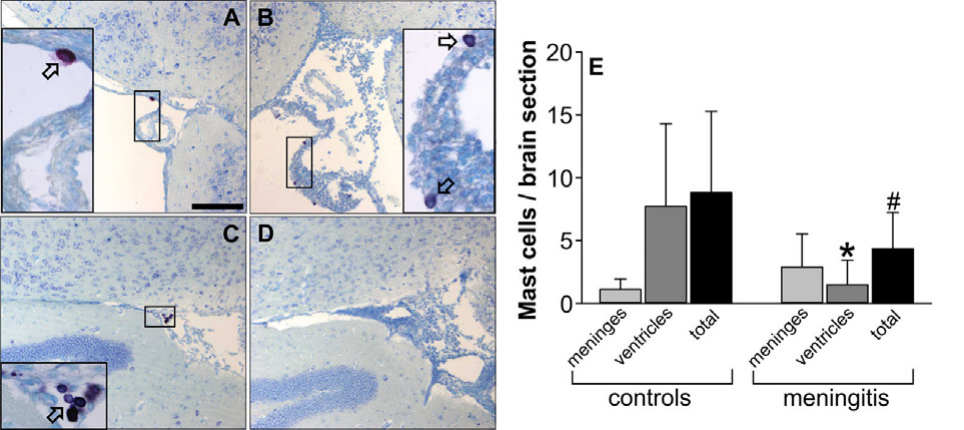
Central nervous system mast cell numbers are reduced during murine pneumococcal meningitis. The images show representative toluidine blue-stained brain sections from control mice **(A,C)** and mice subjected to pneumococcal meningitis **(B,D)**. Images **(A,B)** show cutouts of the leptomeningeal space, whereas **(C,D)** show cutouts of the ventricles. Mast cells stain purple with toluidine blue; arrows indicate representative mast cells. The number of toluidine blue-positive cells was determined in 10 brain sections containing lateral ventricles and the dentate gyrus (thereby focusing on the leptomeningeal space and ventricles) and given as *n* per brain section **(E)**. Scale bars: 100 µm. Data are given as means ± SD. *P < 0.05, compared with the number of mast cells within the ventricles in control mice; ^#^*P* < 0.05, compared with the total number of mast cells in control mice, using Mann–Whitney rank sum test.

To investigate whether the CNS mast cell population is affected by pneumococcal meningitis, we counted the number of metachromatically stained mast cells within the leptomeningeal space and lateral ventricles in brain sections obtained from control and infected mice. Compared with control mice, mice subjected to pneumococcal meningitis showed lower numbers of metachromatic cells within the CNS (especially within the lateral ventricles) at 18 h post infection, suggesting the occurrence of mast cell degranulation and/or death during pneumococcal meningitis (Figure 3).

### WBB6F1-KitW/W^v^ Mice Are Fully Susceptible to Pneumococcal Meningitis

**Figure 4:**
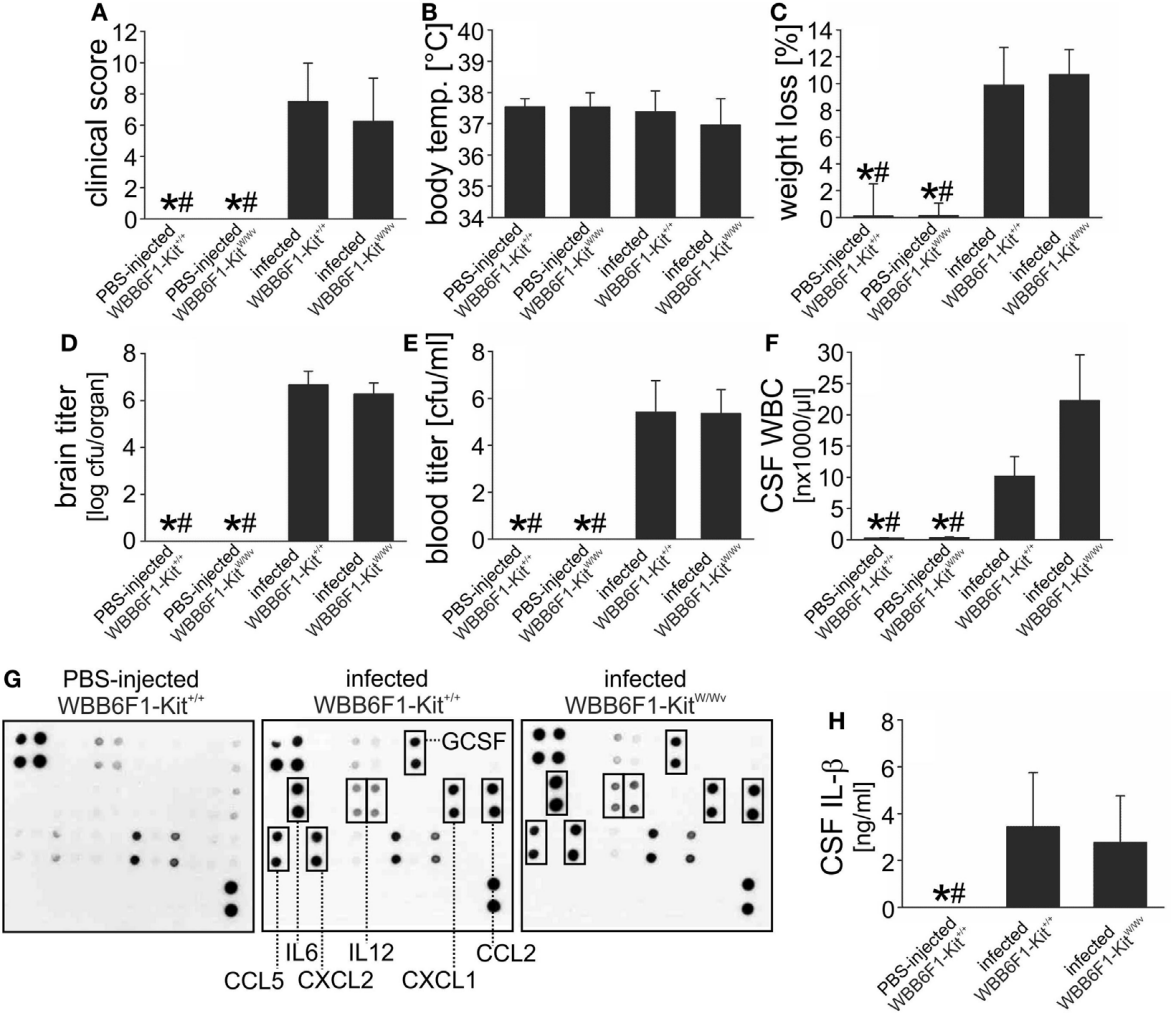
WBB6F1-Kit^W/Wv^ mice are fully susceptible to pneumococcal meningitis. Pneumococcal meningitis was induced by intracisternal injection of *Streptococcus pneumoniae*. Eighteen hours later, animals were evaluated. Except of increased cerebrospinal fluid (CSF) white blood cell (WBC) counts **(F)**, WBB6F1-Kit^W/Wv^ mice showed a similar disease phenotype as WBB6F1-Kit^+/+^ mice, as evidenced by similar clinical score values **(A)**, body temperatures **(B)**, weight losses **(C)**, and brain and blood bacterial titers **(D,E)**. The increase in CSF WBC counts was not associated with increased expression of inflammatory mediators within the central nervous system, as shown by protein array analyses on brain homogenates **(G)** and interleukin (IL)-1β ELISA on CSF samples **(H)**. Data are given as means ± SD. *P < 0.05, compared with infected WBB6F1-Kit^+/+^ mice; ^#^*P* < 0.05, compared with infected WBB6F1-Kit^W/Wv^ mice, using ANOVA and Student–Newman–Keuls *post hoc* test.

The vast majority of the existing data on the role of mast cells in (infectious) diseases is based on experiments in WBB6F1-Kit^W/Wv^ mice (26). WBB6F1-Kit^W/Wv^ mice were used in the only study so far assessing the role of mast cells in an experimental model of brain infection (with Sindbis virus) (31), and exhibited an attenuated immune response and impaired viral clearance. In our murine pneumococcal meningitis model, however, WBB6F1-Kit^W/Wv^ developed disease comparable to their WT littermates WBB6F1-*Kit*^+/+^: The clinical scores, body temperatures, and the meningitis-associated losses in body weight did not differ significantly between both mouse strains (Figures 4A–C). Since mast cells (from human lungs) were reported to exhibit direct antimicrobial activity to *S. pneumoniae* (11), we also determined bacterial loads in brain and blood samples 18 h post infection, by plating of the samples and quantification of cfu. The analysis revealed no significant differences in the amounts of *S. pneumoniae* when comparing mast cell-sufficient and -deficient mice (Figures 4D,E). However, significantly higher leukocyte numbers were found in the CSF of infected WBB6F1-Kit^W/Wv^ mice as compared with WT littermates (Figure 4F). This finding prompted us to analyze the expression of cytokines associated with host inflammatory responses. To get an exhaustive survey, inflammatory cytokine and receptor expression was determined in RNA isolated from frozen brain sections of control mice, infected WBB6F1-*Kit*^+/+^ and WBB6F1-Kit^W/Wv^ mice. The expression of over 20 cytokines, chemokines, and related inflammatory factors was more than twofold upregulated or induced in brains from infected, mast cell-sufficient mice, including IL-1β, IL-6, TNF-α, CXCL2, CXCL10, CCL3, and CCL4 (for more detailed information see Figures S1A,B in Supplementary Material). The expression level of all these factors did not differ substantially between brains from infected mast cell-sufficient and mast cell-deficient mice. In line with these findings, quantitative RT-PCR analysis of IL-1β and IL-33 mRNA brain expression showed similar levels in both mouse strains (Figures S1C,D in Supplementary Material). The finding that brain inflammation was equal in WBB6F1-Kit^+/+^ and WBB6F1-Kit^W/Wv^ mice was further strengthened by cytokine antibody array analysis. Compared with the cytokine protein expression in control brains, 7 out of 32 cytokines were induced or upregulated in brains of infected WBB6F1-Kit^+/+^ mice. Among them were the cytokines IL-6, IL-12, and G-CSF and the chemokines CXCL1, CXCL2, CCL2, and CCL5 (Figures 4E–G). There were no differences in the expression levels of all these factors between infected WBB6F1-Kit^+/+^ and WBB6F1-Kit^W/Wv^ mice. Moreover, ELISA measurements on CSF samples revealed similar increases in IL-1β concentrations in both groups as compared with control mice (Figure 4H). All in all, except for a more pronounced CSF pleocytosis, mast cell-deficient WBB6F1-Kit^W/Wv^ mice showed a similar disease phenotype as their mast cell-sufficient littermates.

### Neither Mast Cell Deficiency due to the Kit^W-sh/KitW-sh^ Mutation nor Pharmacologic Mast Cell Stabilization Influences the Disease Phenotype

**Figure 5:**
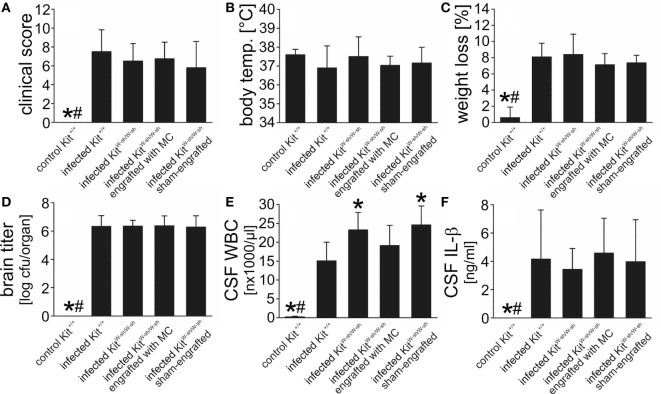
C57BL/6 *Kit*^W-sh/W-sh^ mice show a similar disease phenotype of pneumococcal meningitis than their respective wild-type (WT) littermates. Pneumococcal meningitis was induced by intracisternal injection of *Streptococcus pneumoniae*. Eighteen hours later, animals were evaluated. Except of increased cerebrospinal fluid (CSF) white blood cell (WBC) counts, C57BL/6 *Kit*^W-sh/W-sh^ mice showed a similar disease phenotype as their WT controls C57BL/6 *Kit*^+/+^ mice, as evidenced by similar clinical score values **(A)**, body temperatures **(B)**, weight losses **(C)**, and brain bacterial titers **(D)**. The increase in CSF WBC counts **(E)** was not associated with increased CSF concentrations of interleukin (IL)-1β, as shown by ELISA analysis **(F)**. When injected intracisternally with phosphate-buffered saline, C57BL/6 *Kit*^W-sh/W-sh^ mice had CSF WBC numbers (0.33 ± 0.28 × 10^3^ cells/μl) equal to that of C57BL/6 *Kit*^+/+^ mice. Data are given as means ± SD. *P < 0.05, compared with infected C57BL/6 *Kit*^+/+^ mice; ^#^*P* < 0.05, compared with infected C57BL/6 *Kit*^W-sh/W-sh^ mice, using ANOVA and Student–Newman–Keuls *post hoc* test.

In addition to their mast cell defects, Kit^W/Wv^ mice have multiple hematopoietic abnormalities including compromised fitness of hematopoietic stem and progenitor cells, and macrocytic anemia (26). Importantly, they are neutropenic which may be a major factor affecting immune responses in this strain (26, 55). Owing to these limitations, we additionally evaluated the disease phenotype in another mast cell-deficient, *Kit* mutant mouse strain (C57BL/6 *Kit*^W-sh/W-sh^ mice) as well as in WT mice following treatment with the mast cell stabilizer cromoglycate. The disease phenotype of C57BL/6 *Kit*^W-sh/W-sh^ mice matched that of WBB6F1-Kit^W/Wv^ mice: the clinical status, the body temperature, the loss of body weight, as well as the bacterial concentrations in the brain and blood (not shown) did not differ from that of infected C57BL/6 *Kit*^+/+^ mice (Figures 5A–D). Also similar to WBB6F1-Kit^W/Wv^ mice, infected *Kit*^W-sh/W-sh^ mice exhibited significantly higher CSF leukocyte numbers than the respective controls whereas CSF IL-β levels were quite similar between both mouse strains (Figures 5E,F). To evaluate whether the more pronounced CSF pleocytosis could be reversed by selective repair of the mast cell deficiency, mast cell-deficient *Kit*^W-sh/W-sh^ mice were engrafted i.c. with BMMC from C57BL/6 *Kit*^+/+^ mice. The success of mast cell engraftment was assessed by toluidine blue histological staining of brain sections obtained from infected *Kit*^+/+^, *Kit*^W-sh/W-sh^, and engrafted *Kit*^W-sh/W-sh^ mice: meningeal mast cell numbers were comparable between *Kit*^+/+^ and engrafted *Kit*^W-sh/W-sh^ mice (3.7 ± 1.5 and 3.1 ± 1.9 cells/brain section, respectively), whereas ventricular mast cell numbers tended to be lower in engrafted *Kit*^W-sh/W-sh^ mice than in *Kit*^+/+^ mice (2.0 ± 1.5 and 1.2 ± 1.1 cells/brain section, respectively). No mast cells were detectable in brain sections from *Kit*^W-sh/W-sh^ mice. Although quite successful, mast cell engraftment resulted only in a partial reversal of CSF leukocyte counts to WT levels (of note, leukocyte counts were determined in CSF samples obtained by puncture of the cisterna magna, that is, the meningeal compartment), suggesting that mast cell-independent effects of the *Kit* mutation contribute to this phenomenon. Therefore, as a *Kit* mutation-independent approach, we examined the effect of the well-known mast cell stabilizer cromoglycate in our meningitis model (40, 56, 57). *In vitro*, we found that cromoglycate (when given in a concentration of 10 mM) was capable of blocking cytokine production and degranulation of murine BMMC upon exposure with *S. pneumoniae* (Figures S2A–D in Supplementary Material). *In vivo*, cromoglycate was given directly into the CSF to guarantee that the drug reaches the targeted cells. Treatment with cromoglycate was ineffective in modulating the disease phenotype in the mouse meningitis model: cromoglycate-treated mice had similar clinical score values (6.2 ± 3.1 in cromoglycate-treated mice and 5.9 ± 3.5 in vehicle-treated mice), brain bacterial titers (6.3 ± 0.5 and 6.4 ± 0.7 log cfu/organ, respectively), as well as CSF leukocyte counts (16,271 ± 4,407 and 14,138 ± 4,529 cells/μl, respectively) as vehicle-treated control mice. All in all, our data suggest that mast cells are dispensable for the combat of *S. pneumoniae* infection inside the CSF.

## Discussion

Mast cells are present in the meninges and choroid plexus, where they reside in the vicinity of small vessels. They are, therefore, ideally placed to participate in early pathogen recognition. *In vitro*, rat and human mast cells respond to infection with serotype 2 *S. pneumoniae* by releasing selected immune factors (10, 58). Here, we extend this finding and show that (i) mouse BMMC degranulate and release IL-6, CCL2, CCL3, and CCL4 (but not, for instance, IL-1β and TNF-α) upon exposure to the same infectious agent, (ii) the response of BMMC varies between different pneumococcal serotypes and (iii) is dependent on PLY, but independent on TLR activation. Based on these findings, we anticipated that the absence of mast cells or pharmacologic mast cell stabilization (by cromoglycate) may reduce inflammation and ameliorate disease severity following intracisternal infection of mice with *S. pneumoniae*. Intriguingly though, apart from a slight enhancement of CSF pleocytosis, we did neither observe any effects of mast cell deficiency on brain cytokine and chemokine expression nor on disease severity. In addition, neither mast cell deficiency nor cromoglycate treatment had any impact on pneumococcal growth and dissemination in pneumococcal meningitis.

There is now a wealth of evidence suggesting that mast cells are crucial players in the host defense toward bacterial insults. Mast cells can modulate host innate immunity by releasing preformed and/or newly synthetized mediators (13). Their release is triggered through recognition of bacteria or bacterial products by various PRRs expressed on the cell surface or within endocytic vehicles (14) and, depending on the activating signal, exhibits distinct dynamics and features that are associated with distinct inflammation patterns (14, 59). Accordingly, we detected substantial differences in the BMMC reaction to different pneumococcal serotypes: while challenge with a serotype 2 strain caused both, mast cell degranulation and cytokine release, stimulation with a serotype 19A strain only triggered cytokine secretion, and exposure to serotype 3 or 7F strains was even ineffective. A possible explanation for this finding may be that the serotypes differ in their expression of the cholesterol-dependent cytolysin PLY, as demonstrated by Western immunoblotting and a standard hemolysis test (Figures S3A,B in Supplementary Material). PLY seems to be important for mast cell activation upon *S. pneumoniae* infection as it can stimulate human lung mast cells to release the antimicrobial peptide LL-37 and the pro-inflammatory mediator LTC_4_ (11). Accordingly, we found that, in the serotype 2 strain, genetic deletion of PLY results in complete loss of its ability to activate BMMC. The mechanism through which PLY activates mast cells is not known. Based on previous reports that PLY can activate TLR4 (60) and that mast cells can express most of the common TLR including TLR1–9 (14), TLR involvement has been speculated (11). By using pharmacologic TLR antagonists and BMMC from TLR-deficient mice, however, we were unable to find evidence for a linkage between TLR and *S. pneumoniae*-induced BMMC. Of note, these experiments were hampered by possible pharmacologic side effects of chloroquine (51, 52) and by an incomplete differentiation of *MyD88/TRIF^−/−^* bone marrow into mast cells, as evidenced by FACS immunophenotyping for the mast cell marker c-Kit and Fc-epsilon receptor I. Also of note, our observation that BMMC can release selected cytokines (namely, IL-6, CCL2, CCL3, and CCL4) in response to pneumococcal challenge is in contradiction to previous studies: Barbuti et al. (10) failed to show IL-6 and TNF-α production in the rat mast cell line RBL-2H3, and Cruse et al. (11) did not detect synthesis of several cytokines (including IL-6 and CCL2) in human lung mast cells. These striking differences might be explained by the following facts: (i) different activation signals can trigger distinct mast cell responses (30, 59). In our study, mast cells were stimulated with antibiotic-lysed bacteria, whereas live bacteria (albeit the same strain) and recombinant PLY were used in the other studies, respectively (10, 11). (ii) Mast cells exhibit a high degree of phenotypic heterogeneity and plasticity (61). In our study, experiments were conducted in mouse bone marrow cells differentiated into mast cells by propagation over 4–6 weeks in the presence of IL-3 and SCF, whereas rat or human mast cell lines as well as primary human mast cells which were purified from macroscopically normal lung tissue obtained from cancer resections were employed in the other studies. Taken together, the existing data indicate that mast cells are capable of reacting to *S. pneumoniae* infection. However, the response pattern likely varies between different stimuli and mast cell populations.

Intriguingly, although we found *in vitro* and histological evidence for mast cell activation upon *S. pneumoniae* exposure, we did not see any effect of mast cell deficiency (by using two different *Kit* mutant mouse strains) on the disease phenotype, bacterial outgrowth, and CNS inflammation (except for an increase in CSF pleocytosis) in an *in vivo* meningitis model. One potential explanation for this discrepancy may be that although brain mast cells may respond to CSF *S. pneumoniae* infection with the release of cytokines, their total output is negligible in comparison with contributions from other cells, e.g., resident macrophages. In this context it is important to point out that mast cells represent only a minor population of cells with the leptomeningeal space (the site of infection in meningitis) (7, 8). Another possible explanation for the apparent differences between the cytokine responses seen *in vitro* and *in vivo* is that the mast cell phenotype and consequently their response pattern may differ between the *in vitro* situation and *in vivo* (61). Culture of bone marrow-derived progenitor mast cells with IL-3 and SCF facilitates maturation toward a connective-tissue type phenotype (62, 63). Although CNS mast cells belong predominantly to this phenotype, there are also other subpopulations including mucosal type and mixed type mast cells present in the meninges and brain parenchyma (41, 64), illustrating substantial mast cell heterogeneity within the CNS. In addition, the mast cell heterogeneity can probably dynamically change in accordance with microenvironmental conditions (e.g., alterations in cytokine levels, cell–cell contacts, etc.) that dictate their gene expression and phenotypic development (61). Since the microenvironmental conditions where mast cell phenotypic development occurs *in vivo* cannot be fully mimicked *in vitro*, cultured mast cells are inevitably incomplete representatives of mature tissue mast cells.

Despite similar cytokine production profiles, *Kit* mutant mice exhibited significantly higher leukocyte (particularly neutrophil) counts in the CSF 18 h after pneumococcal infection as compared with mast cell-sufficient control mice. Our observation is in conflict with the long time widely accepted theory that mast cells contribute to neutrophil recruitment in infectious diseases as initially reported by Echtenacher and Malaviya and their respective coworkers (15, 16). Accordingly, a more recent study in a mouse model of pneumococcal pneumonia demonstrated a reduction of leukocyte recruitment into lung tissue during established disease in mast cell-deficient *Kit*^W-sh/W-sh^ mice (12). Although the exact reasons for this disparate findings remain elusive, evidence exists that (i) the innate immune responses to *S. pneumoniae* infection differ between the lung and CNS (65, 66) and (ii) mast cells can have no effect on, promote or block leukocyte infiltration, depending on the mouse strain background, the nature of the mutation resulting in a mast cell deficiency, and type and severity of infection (25, 26). Furthermore, it is worth mentioning that i.c. mast cell reconstitution only partially reverses CSF leukocyte counts to values seen in infected mast cell-sufficient mice. Local mast cell reconstitution (in mast cell-deficient *Kit* mutant mouse strains) is considered as the mainstay to study mast cell function *in vivo*. Although engrafted BMMC can adopt the phenotype of normal tissue mast cells after transfer, numbers, distribution, and functional responses of reconstituted mast cells may not be physiological (67, 68). Hence, it seems not possible to define the exact contribution of mast cells (or to be precise their deficiency) to the increase in CSF pleocytosis observed in our study. Nonetheless, we think that its incomplete reversal by mast cell engraftment may point at other c-Kit mutation-related mechanisms (whose identification was beyond the scope of this study) than mast cell deficiency. This hypothesis is supported by our observation that i.c. treatment with the mast cell stabilizer cromoglyate showed no effect on leukocyte recruitment into the CSF in response to *S. pneumoniae* infection.

Mast cells can also participate in direct killing of pathogens by phagocytosis, extracellular trap formation, and/or the release of antimicrobial peptides, such as the cathelicidin LL-37 (13, 14). Accordingly, human lung mast cells were reported to exhibit direct antimicrobial activity to *S. pneumoniae* upon activation by the bacterial toxin PLY. This antimicrobial activity is mediated, at least partly, by the secretion of LL-37 from mast cells (11). Paradoxically, in a mouse model of pneumococcal pneumonia, mast cell deficiency did not significantly influence the clearance of PLY-sufficient *S. pneumoniae* strain, whereas its isogenic PLY-deficient mutant was cleared more rapidly in mast cell-deficient mice (*Kit*^W-sh/W-sh^ mice), suggesting even an improved host defense against pneumococcal infection in mice with profound mast cell deficiency. Contrary to both studies, we did not detect any differences in bacterial loads in the brain and blood between mast cell-sufficient and mast cell-deficient *Kit* mouse mutant strains as well as vehicle- or cromoglycate-treated mice. This observation may be related to the well-known immune incompetence inside the CSF-filled leptomeningeal space, such as a lack of complement factors, hampering antibacterial effector mechanisms (2).

In conclusion, we have shown that (i) mouse BMMC degranulate and release selected cytokines upon exposure to *S. pneumoniae*, (ii) the response of BMMC varies between different pneumococcal serotypes and (iii) is dependent on PLY. Intriguingly though, we did neither observe any effects of mast cell deficiency nor of pharmacologic mast cell stabilization on the disease phenotype in a well-established mouse model of pneumococcal meningitis. Hence, mast cells are unlikely to act as sentinels of pneumococcal CSF invasion and initiators of the host immune response inside the CSF.

## Ethics Statement

All procedures were approved by the Committee on the Ethics of Animal Experiments of the Government of Upper Bavaria (Permit numbers 55.2-1-54-2531-67-99, -125-13) and carried out in accordance with the Principles of Laboratory Animal Care (European Commission Directive 2010/63/EU), the German Animal Welfare Act, and the ARRIVE Guidelines.

## Author Contributions

JF carried out the cell culture experiments and participated in the *in vivo* study. UK, JB, and MK performed the mouse experiments, with the assistance of SD, IM, and SV (clinical monitoring throughout the experiment). IM and SV carried out the histological analyses on mouse brain samples (from sample preparation to statistical analysis). DA and HS carried out the immunophenotyping experiments. SH provided the pneumococcal strains. UK, SH, and HS facilitated the lab utilities and provided technical and substantive advices. H-WP, MK, and UK designed the study. The manuscript was drafted by UK and MK and discussed and edited by SD, SH, HS, and H-WP. All the authors read and approved the final manuscript.

## Conflict of Interest Statement

Herewith, the authors declare that the research was conducted in the absence of any commercial or financial relationships that could be construed as a potential conflict of interest.

## Footnotes

^1^www.raybiotech.com.

^2^www.superarray.com.

## Supplementary Material

Figure S1 Effect of mast cell deficiency on cytokine mRNA expression within the brain following intracisternal pneumococcal infection. **(A)** A mouse inflammatory cytokines and receptors oligonucleotide array was used to determine the differences in the mRNA expression of cytokines, chemokines, and other inflammatory factors from PBS-injected WBB6F1-*Kit*^+/+^ controls (left image) as well as Streptococcus pneumoniae-infected WBB6F1-*Kit*^+/+^ mice (middle image) and WBB6F1-*Kit*^W/Wv^ mice (right image). The expression of multiple cytokines, chemokines, and related inflammatory factors was upregulated or induced in brains from infected WBB6F1-*Kit*^+/+^ mice. The expression level of all these factors did not differ between brains from infected WBB6F1-*Kit*^+/+^ and WBB6F1-*Kit*^W/Wv^ mice. **(B)** A scheme of the oligonucleotide array membrane: inflammatory factors that were upregulated more than fourfold or clearly induced in infected mice are marked with yellow. **(C)** Using mRNA isolated from mouse brains, the relative expressions of IL-1β and IL-33 were analyzed in comparison to the housekeeping gene aldolase using quantitative RT-PCR. Data are given as mean ± SD. **P* < 0.05, compared to infected WBB6F1-*Kit*^+/+^ mice, #*P* < 0.05, compared to infected WBB6F1-*Kit*^W/Wv^ mice, using ANOVA and Student–Newman–Keuls test for post hoc analysis.

Figure S2 Effect of cromoglycate treatment on the BMMC response to pneumococcal challenge. In order to evaluate the mast cell-inhibitory potential of cromoglycate **(CG)**, we tested the responsiveness of murine BMMC to pneumococcal challenge (10^7^ cfu/ml) in the absence or presence of different CG concentrations. Supernatants were collected 6 h after challenge, and IL-6 **(A)**, CCL2 **(B)**, β-hexosaminadase **(C)**, and LDH **(D)** concentrations were analyzed by standard assays. All experiments were repeated thrice in triplicates. Data are given as mean ± SD. **P* < 0.05, compared to vehicle (=PBS)-treated, stimulated BMMC, #*P* < 0.05, compared to unstimulated BMMC, using ANOVA and Student–Newman–Keuls test for post hoc analysis.

Figure S3 Analysis of pneumolysin production by Streptococcus pneumoniae serotypes. Pneumolysin production of the each pneumococcal strain was tested using a standard hemolysis assay **(A)** and Western blot analysis **(B)**. The presence of pneumolysin is indicated by disintegration of the red blood cell pellet within the well in the hemolysis assay and by a single band of approximately 53 kDa in the Western blot analysis. Antibiotic-lysed S. pneumoniae of the serotypes 2 (pneumolysin-sufficient or -deficient strain), 3, 7F, and 19A were used as samples in both investigations.
