# Supplementary figures and images for: Mast Cells Are Activated by Streptococcus pneumoniae In Vitro but Dispensable for the Host Defense Against Pneumococcal Central Nervous System Infection In Vivo
Source: Front Immunol. 2018 Mar 19;9:550. doi: 10.3389/fimmu.2018.00550 (PMC5867309; doi:10.3389/fimmu.2018.00550)

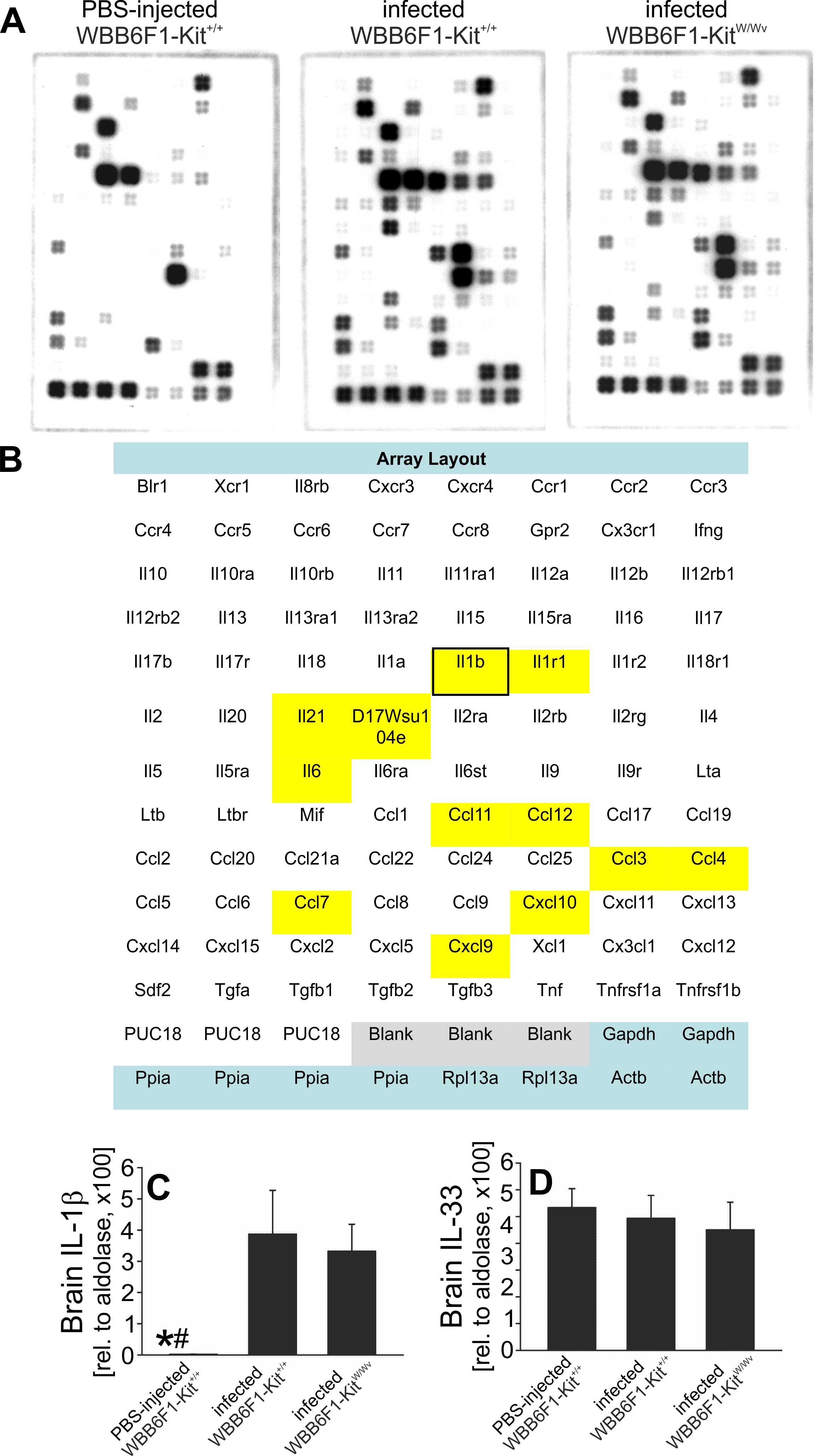

Supplement: Figure S1 — Effect of mast cell deficiency on cytokine mRNA expression within the brain following intracisternal pneumococcal infection. (A) A mouse inflammatory cytokines and receptors oligonucleotide array was used to determine the differences in the mRNA expression of cytokines, chemokines, and other inflammatory factors from PBS-injected WBB6F1-Kit+/+ controls (left image) as well as Streptococcus pneumoniae-infected WBB6F1-Kit+/+ mice (middle image) and WBB6F1-KitW/Wv mice (right image). The expression of multiple cytokines, chemokines, and related inflammatory factors was upregulated or induced in brains from infected WBB6F1-Kit+/+ mice. The expression level of all these factors did not differ between brains from infected WBB6F1-Kit+/+ and WBB6F1-KitW/Wv mice. (B) A scheme of the oligonucleotide array membrane: inflammatory factors that were upregulated more than fourfold or clearly induced in infected mice are marked with yellow. (C) Using mRNA isolated from mouse brains, the relative expressions of IL-1β and IL-33 were analyzed in comparison to the housekeeping gene aldolase using quantitative RT-PCR. Data are given as mean ± SD. *P < 0.05, compared to infected WBB6F1-Kit+/+ mice, #P < 0.05, compared to infected WBB6F1-KitW/Wv mice, using ANOVA and Student–Newman–Keuls test for post hoc analysis. [file Image_1.JPEG]

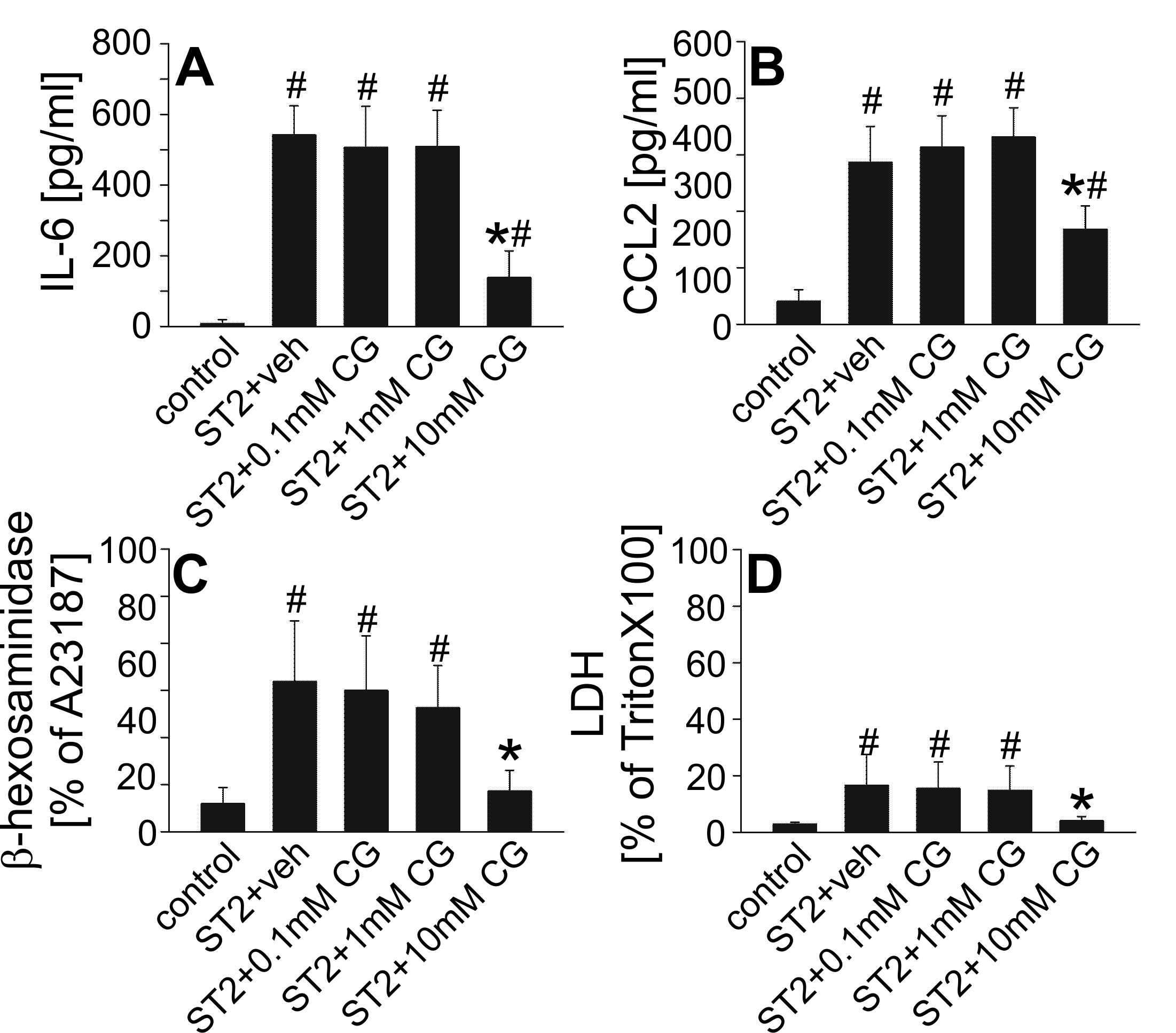

Supplement: Figure S2 — Effect of cromoglycate treatment on the BMMC response to pneumococcal challenge. In order to evaluate the mast cell-inhibitory potential of cromoglycate (CG), we tested the responsiveness of murine BMMC to pneumococcal challenge (107 cfu/ml) in the absence or presence of different CG concentrations. Supernatants were collected 6 h after challenge, and IL-6 (A), CCL2 (B), β-hexosaminadase (C), and LDH (D) concentrations were analyzed by standard assays. All experiments were repeated thrice in triplicates. Data are given as mean ± SD. *P < 0.05, compared to vehicle (=PBS)-treated, stimulated BMMC, #P < 0.05, compared to unstimulated BMMC, using ANOVA and Student–Newman–Keuls test for post hoc analysis. [file Image_2.JPEG]

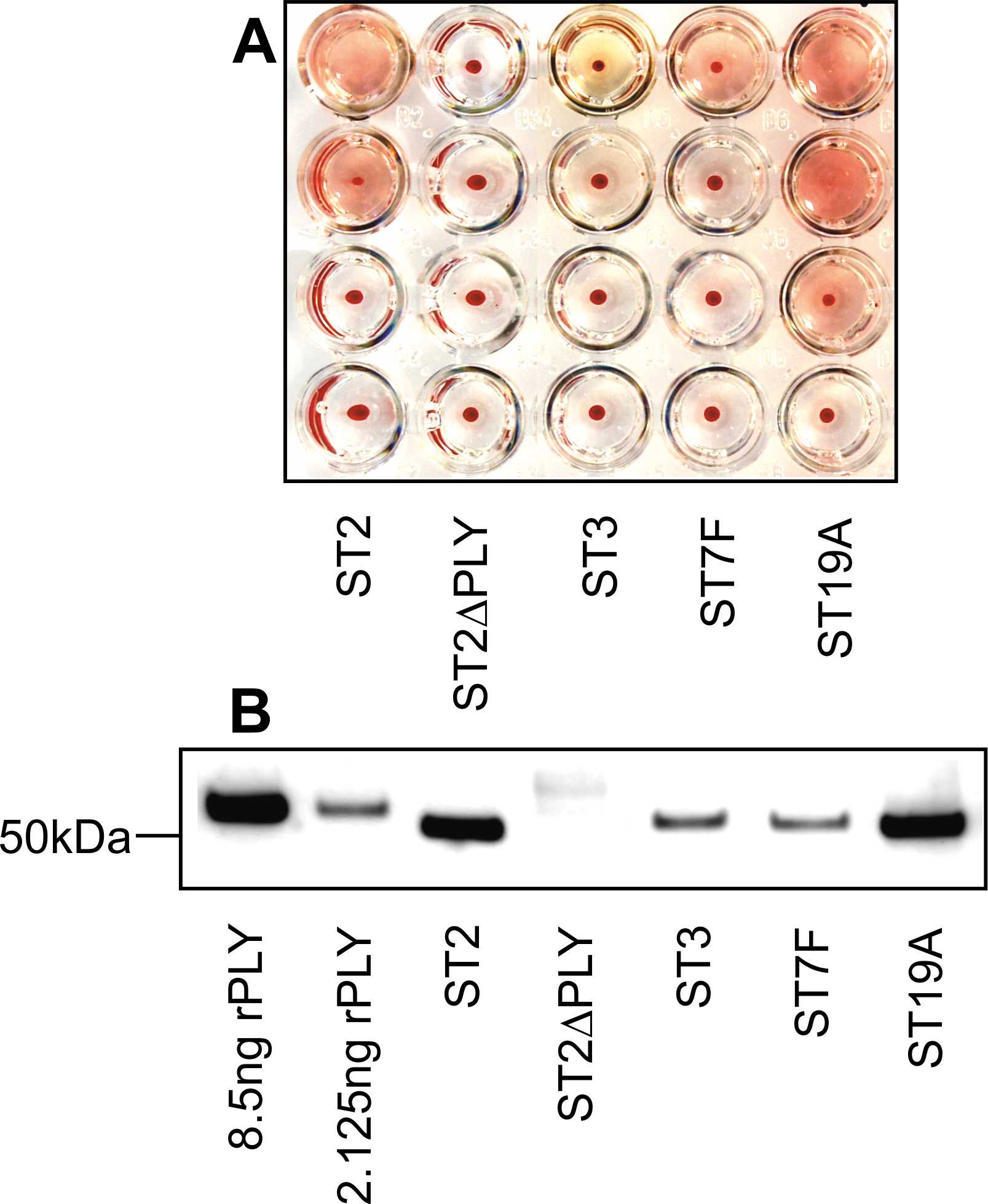

Supplement: Figure S3 — Analysis of pneumolysin production by Streptococcus pneumoniae serotypes. Pneumolysin production of the each pneumococcal strain was tested using a standard hemolysis assay (A) and Western blot analysis (B). The presence of pneumolysin is indicated by disintegration of the red blood cell pellet within the well in the hemolysis assay and by a single band of approximately 53 kDa in the Western blot analysis. Antibiotic-lysed S. pneumoniae of the serotypes 2 (pneumolysin-sufficient or -deficient strain), 3, 7F, and 19A were used as samples in both investigations. [file Image_3.JPEG]
